# The Impact of Emotional Intelligence on Nurses' Professional Quality of Life in Pre‐Hospital Emergency Settings: A Multicentre Mixed‐Method Study

**DOI:** 10.1111/jocn.17511

**Published:** 2024-10-20

**Authors:** Maria Emma Musio, Francesca Ginogi, Simone Casini, Graziana Lucente, Fiona Timmins, Mark Hayter, Gianluca Catania, Milko Zanini, Giuseppe Aleo, Loredana Sasso, Annamaria Bagnasco

**Affiliations:** ^1^ Department of Health Sciences University of Genoa Genoa Italy; ^2^ School of Nursing, Midwifery & Health Systems University College Dublin Dublin Ireland; ^3^ Faculty of Health and Education Manchester Metropolitan University Manchester UK

**Keywords:** emotional intelligence, pre‐hospital emergency nurse, professional quality of life

## Abstract

**Background:**

Pre‐hospital emergency nurses, frequently exposed to high‐stress situations, are at risk for burnout and stress‐related issues, affecting their overall well‐being. The Professional Quality of Life (ProQoL) scale, widely used among hospital nurses, remains untested in pre‐hospital emergency settings.

**Aim:**

To adapt and validate the ProQoL scale for pre‐hospital emergency contexts and explore the protective role of emotional intelligence in professional well‐being.

**Methods:**

A mixed‐method study was conducted. The qualitative approach involved semi‐structured interviews to inform the modification of items for adapting the ProQoL to the pre‐hospital emergency setting. A quantitative method was applied to assess the relationship between emotional intelligence and professional well‐being through content and face validity measures.

**Results:**

Qualitative interviews suggested refining the ProQoL for pre‐hospital emergency settings, emphasising factors such as job satisfaction and professional conduct. The revised 21‐item Pre‐Hospital Emergency‐Professional Quality of Life (PHE‐ProQoL) scale demonstrated strong content validity (I‐CVI: 0.86‐1, S‐CVI: 0.9) and face validity. Significant correlations were observed between emotional intelligence and professional well‐being, with negative correlations between emotional intelligence and both burnout (Pearson's *r* = −0.859) and post‐traumatic stress (Pearson's *r* = −0.792), and a positive correlation with compassion satisfaction (Pearson's *r* = +0.917). Pre‐hospital nurses displayed moderate levels of compassion satisfaction (27.3 ± 9.81), high emotional intelligence (28.0 ± 9.58), especially in empathy, and substantial levels of burnout (22.5 ± 6.09) and stress (21.2 ± 4.3).

**Discussion:**

The study found that pre‐hospital emergency nurses exhibit moderate compassion satisfaction and above‐average emotional intelligence, particularly in perceiving and managing others' emotions. However, they also experience significant levels of burnout and post‐traumatic stress.

**Conclusions:**

Burnout and post‐traumatic stress significantly affect pre‐hospital emergency nurses. Enhancing emotional intelligence is crucial for their well‐being. Nursing managers now have access to a validated and reliable tool to assess this.

**Summary**

- This paper validates the ProQoL scale for use in pre‐hospital emergency settings, offering a reliable tool for assessing well‐being, burnout and stress among pre‐hospital emergency nurses.
- It highlights the critical role of emotional intelligence in mitigating burnout and stress while enhancing compassion satisfaction, offering insights for interventions aimed at improving professional well‐being.
- The study provides practical implications for nursing managers by introducing a refined and validated 21‐item PHE‐ProQoL scale that can be used globally to enhance the well‐being of emergency nurses, potentially leading to better patient care and nurse retention.

## 1 Background

The roles of paramedics and pre‐hospital emergency nurses are shaped by various factors, including healthcare systems, regulations and cultural norms, which vary significantly across countries. Paramedics typically receive specialised training ranging from vocational certificates to bachelor's degrees, with their scope of practice encompassing advanced emergency interventions such as medication administration, intubation and advanced cardiac life support (Peitzman and Sarani 2010). In contrast, pre‐hospital emergency nurses, who are registered nurses with additional training in emergency care, are more prevalent in integrated healthcare systems and possess a broader medical knowledge base, although they may face legal restrictions on certain interventions (Mota, Cunha, and Santos 2020).

While emergency nurses and paramedics share essential roles in providing critical care, they differ significantly in several aspects. Emergency nurses typically undergo a more extensive and comprehensive educational process, ranging from 2 to 4 years, leading to an Associate Degree in Nursing (ADN) or a Bachelor of Science in Nursing (BSN). This education covers various subjects, including anatomy, physiology, pharmacology and nursing theory, and includes supervised clinical practice in diverse healthcare settings (Benner 2001). In contrast, paramedics usually complete shorter, specialised training programmes that focus primarily on emergency medical care, such as advanced life support and trauma management, lasting 1–2 years (Carter and Thompson 2020).

The scope of practice also differs. Emergency nurses are trained to provide a broad range of care that extends beyond emergency interventions to include long‐term patient management, health education and disease prevention across various settings, such as hospitals, clinics and community health environments (American Nurses Association 2020). Conversely, paramedics specialise in delivering immediate, life‐saving care in pre‐hospital settings, focusing on stabilising patients in life‐threatening situations and ensuring their safe transport to healthcare facilities. Their role typically concludes once the patient reaches the hospital, reflecting a more focused and time‐limited scope of practice (Kane, Haigh, and Wilson 2019).

Differences in work environments further highlight their distinct roles. Emergency nurses generally work in controlled settings, such as hospitals or clinics, where they have access to various resources and can provide coordinated and holistic care (American Nurses Association 2020). In contrast, paramedics operate primarily in the field, often under unpredictable and challenging conditions, such as at the scene of an accident or in a patient's home, where they must provide emergency care with limited resources (Carter and Thompson 2020).

Another difference lies in the nature of patient interactions. Emergency nurses typically develop longer‐term, continuous relationships with patients, providing care throughout the healthcare journey from admission to discharge, which allows for deeper connections and more personalised, patient‐centred care (Benner 2001). In contrast, paramedics usually engage in brief, high‐intensity interactions with patients, focusing on rapid assessment and immediate interventions to stabilise the patient before transport to a medical facility (Kane, Haigh, and Wilson 2019).

The regulatory frameworks governing these professions also differ. Nursing is regulated by extensive frameworks that require state or national licensure and adherence to strict ethical standards, guiding both independent and collaborative practice (American Nurses Association 2020). Paramedics, while also subject to certification and professional standards, operate primarily under protocols set by their employers and medical directors, which can limit their professional autonomy compared to nurses (Carter and Thompson 2020).

Career progression opportunities further distinguish the two professions. Nursing offers numerous pathways for advancement and specialisation, enabling nurses to pursue advanced degrees and specialised roles such as nurse practitioners, clinical nurse specialists or nurse anaesthetists (Benner 2001). Paramedic careers, however, are generally more limited, with fewer opportunities for specialisation beyond administrative roles, education or related fields like firefighting or emergency management (Kane, Haigh, and Wilson 2019).

Despite these differences, emergency nurses and paramedics share some similarities. Both play crucial roles in emergency care, focusing on stabilising patients and providing critical interventions in acute situations (Peitzman and Sarani 2010; Mota, Cunha, and Santos 2020). They both require proficiency in acute interventions, such as administering medications, performing resuscitations and providing advanced life support (Peitzman and Sarani 2010). Furthermore, both professions frequently collaborate with other healthcare professionals to ensure optimal patient outcomes, demonstrating a shared commitment to patient care in high‐pressure situations (American Nurses Association 2020; Kane, Haigh, and Wilson 2019).

Overall, while the roles of emergency nurses and paramedics differ significantly in terms of education, scope of practice, work environment, patient interaction, regulation and career opportunities, both are essential to the healthcare system, particularly in their shared goal of providing critical care to patients in need.

In Emergency Medical Systems, countries like the United States, Canada and Australia predominantly rely on paramedics, distinguishing between Basic and Advanced Life Support levels. Meanwhile, European regions, especially Scandinavia and France, rely on pre‐hospital emergency nurses for critical and specialised cases (Suzuki et al. 2008). The regulatory environment for these professions varies, with paramedics in some countries facing regulations similar to other healthcare professionals, while nurses generally adhere to more standardised international regulations, though their specific pre‐hospital roles may be less defined (Whetzel and Wagner 2008).

Cultural and systemic influences significantly impact these roles. In hospital‐centric systems, there is a preference for pre‐hospital emergency nurses, while in decentralised systems, paramedics often serve as the primary independent providers. The trend towards interdisciplinary teams is growing, particularly in regions with advanced EMS systems, combining both professions to deliver comprehensive care. These roles continue to evolve due to ongoing research and changing healthcare demands, with innovations in telemedicine, community paramedicine and integrated models influencing their development (Suserud and Haljamäe 1997; Heardman 2014).

The paramedic profession is legally recognised and regulated in many English‐speaking countries, requiring registration with professional boards (e.g., in the UK, Ireland, USA, Canada, Australia and New Zealand). They are known by various titles, such as emergency medical technician, personal emergency medical technician, emergency response personnel and call takers, depending on the region (Lawn et al. 2020). Studies show that paramedics face diverse job stresses, with responses varying individually. This stress can lead to depression, work‐related issues and emotional challenges (Harris et al. 2023; Lawn et al. 2020). The continuous pressure and unpredictability of their work environment are significant stress factors, making stress management vital for both patient care and their well‐being (Poranen, Kouvonen, and Nordquist 2022; Duffee and Willis 2023). Pre‐hospital emergency care, crucial for patient outcomes, requires excellent knowledge and skills, significantly impacting patient quality of life (Abate and Mekonnen 2020).

This overview underscores the diversity and adaptability of the roles of paramedics and pre‐hospital nurses, shaped by the unique requirements of emergency medical care globally, and shows how the continuous evolution of these professions is critical to meeting the dynamic demands of pre‐hospital emergency care worldwide. In this context, emotional intelligence (EI), that encompasses the ability to control one's emotions and those of others, emerges as a predictor of professional success, highlighting its importance for professionals in emergency situations (Jiménez‐Picón et al. 2021).

Research shows that EI positively influences occupational well‐being. Emotionally intelligent individuals are better able to cope in the workplace (Molero Jurado et al. 2019); emotional intelligence is considered a key skill for nurses to interact respectfully with patients (Rego et al. 2010), and positive correlations have been found between EI and performance (Codier et al. 2010). Additionally, the World Health Organization considers EI one of the 10 essential life skills that help people act adaptively and positively (Ruíz 2014).

Healthcare professionals face numerous challenges and must manage multiple stressors in their practice, such as time constraints, inadequate support, and exposure to trauma, morbidity, and mortality. Furthermore, they often work with limited resources, hindering their ability to deliver the care they aspire to provide. This can lead to burnout, secondary traumatic stress and compassion fatigue, a state that is uncommon in the general population but prevalent among healthcare professionals (Thapa et al. 2021).

International organisations, including the WHO, have noted that certain psychosocial risk factors, such as stress, burnout syndrome and workplace violence, are becoming increasingly relevant in developed countries. Numerous studies have shown that professionals working with persons with disabilities are exposed to many psychosocial factors and experience high levels of stress, but few studies have examined protective factors (Guerrero‐Barona et al. 2020).

The contribution of nurses to global health is undisputed, and investing in improving their quality of life benefits society. Enhancing working conditions and quality of life for nurses not only improves their well‐being but also their performance, benefiting the entire healthcare system (Soto‐Rubio, Giménez‐Espert, and Prado‐Gascó 2020). The Professional Quality of Life (ProQoL) scale (Palestini et al. 2009) is a commonly used measure of compassion fatigue and satisfaction in the nursing literature. The ProQoL is designed as a screening tool for the positive and negative aspects of working in a helping profession, such as nursing.

To be reliably used in the pre‐hospital emergency setting, the ProQoL tool needs adaptation and validation.

Given the unique challenges faced by pre‐hospital emergency nurses, such as high‐stress environments, limited resources and the need for rapid decision‐making, there is a pressing need to explore protective factors that can enhance their professional quality of life. Emotional intelligence (EI) has been identified as a potential key factor in promoting occupational well‐being by helping healthcare professionals manage stress, maintain empathy and improve interpersonal relationships (Molero Jurado et al. 2019; Rego et al. 2010). However, while EI has been widely studied in various healthcare settings, its role in the specific context of pre‐hospital emergency care remains underexplored. Furthermore, existing tools like the ProQoL scale, which are extensively used to assess the well‐being of hospital nurses, have not yet been validated for use in the pre‐hospital emergency environment. This study aims to address these gaps by adapting the ProQoL scale for pre‐hospital emergency nurses and examining the impact of EI on their professional quality of life. By doing so, it seeks to provide a validated tool and new insights that can inform strategies to support and improve the well‐being of these essential healthcare professionals.

## 2 Study Aim

This study aims to determine whether the protective role of EI on the professional well‐being of nurses, as described in the literature for critical care settings, is also applicable in the pre‐hospital emergency setting. To achieve this objective, we adapted and validated the ProQoL scale specifically for use in pre‐hospital emergency environments.

## 3 Methods and Instruments

### 3.1 Study Design

A mixed‐method, multicentre study was conducted to adapt the ProQoL scale for use in a pre‐hospital emergency setting. The study comprised both qualitative and quantitative components. The qualitative component involved semi‐structured interviews with emergency nurses to gather in‐depth insights. These interviews guided the modification of the ProQoL scale items, ensuring relevance and applicability to the specific context of pre‐hospital emergency care.

Simultaneously, a quantitative approach was used to evaluate the relationship between EI and professional well‐being. This was achieved by assessing the content and face validity of the modified ProQoL scale. Content validity was used to ensure that the items on the scale adequately covered all aspects of the constructs being measured, while face validity confirmed that the items appeared relevant and appropriate to the respondents. Together, these methods established a valid and reliable measure for examining how EI influences professional well‐being in the pre‐hospital emergency care environment.

### 3.2 Tool Adaptation to the Pre‐Hospital Emergency Setting and Validation Process

Qualitative data were collected through six semi‐structured interviews with pre‐hospital emergency nurses to explore their concepts of professional well‐being. These data guided the modification of items to adapt the ProQoL scale to the pre‐hospital emergency setting. The six nurses interviewed were selected through convenience sampling.

Quantitative data were collected via an electronic questionnaire sent to pre‐hospital emergency nurses, also recruited through convenience sampling. The content and face validity of the new tool, named Pre‐Hospital Emergency‐Professional Quality of Life (PHE‐ProQoL), were assessed by a panel of seven experts, including four males and three females. These experts, comprising both physicians and nurses actively working in the pre‐hospital emergency sector, had specialised knowledge in personnel management and professional well‐being. They provided valuable insights for the adaptation and validation of the scale.

### 3.3 The Study

#### 3.3.1 Data Collection

Two validated instruments in the Italian language were used: the Brief Emotional Intelligence Scale (Durosini et al. 2021) and the adapted version of the Professional Quality of Life Scale (ProQoL) for the pre‐hospital emergency setting (Palestini et al. 2009), named the PHE‐ProQoL.

The 10‐item short version of the Brief Emotional Intelligence Scale was employed to self‐assess the ability to recognise and manage both personal and others' emotions. The PHE‐ProQoL consists of 21 items that evaluate three key dimensions of professional life: compassion satisfaction, burnout and secondary traumatic stress.

#### 3.3.2 Sample

The study was conducted across four pre‐hospital emergency centres located throughout the Liguria region. A total of 91 pre‐hospital emergency nurses were recruited through convenience sampling, targeting those currently employed in emergency services within Liguria.

Inclusion Criteria:

- Being a nurse.
- Working in the Ligurian pre‐hospital emergency setting.

Exclusion Criteria:

- Nurses serving in settings other than pre‐hospital emergency.

### 3.4 Ethical Considerations

Ethical approval for this study was obtained from the relevant institutional review board, ensuring that all research activities complied with ethical guidelines for research involving human participants. Informed consent was obtained from all participants, and confidentiality was maintained throughout the study. Participants were assured that their responses would be anonymised and used solely for research purposes. The study adhered to ethical standards by minimising risks and ensuring the voluntary nature of participation.

## 4 Results

### 4.1 Adaptation of the Tool

The qualitative component, involving six semi‐structured interviews, guided the adaptation of the ProQoL scale to the pre‐hospital emergency setting. The participants were six nurses with extensive work experience in the pre‐hospital emergency environment, with an average age of 46 ± 12 years and an average of 12 ± 8 years of experience in hospital emergency settings.

The interviews revealed that to be effective in assessing professional well‐being in the pre‐hospital emergency setting, the instrument should investigate specific factors fundamental to professional well‐being, such as a positive work environment, job satisfaction, professional conduct and passion for the profession. Additionally, it should consider factors that can either support or hinder professional well‐being, such as professional development, relational factors, trust among team members, availability of suitable equipment and staffing levels (Table 1).

**TABLE 1:** Themes, subthemes and quotations from semi‐structured interviews.

Pre‐hospital emergency nurses' professional well‐being.	Constituent elements	Passion for the profession	‘…began as a passion […] I travelled around Italy to take courses…’	
		Job satisfaction	‘… be proud of our role…’	
		Positive work environment	‘…the luck of working with a competent team…’	
		Respect for professional conduct	‘…the key thing is respect for the patient…’	
	Hindering and/or favouring factors	Organisational	Staffing levels	‘… the number of working hours affects a lot…’
			Feedback systems on intervention and patient outcomes	‘…a patient is aided and after that we know nothing…’
		Management	Availability of suitable equipment	‘…we are very limited in terms of materials and equipment…’
		Empowerment	Involvement in organisational choices	‘…we don't always have the opportunity to express ourselves…’
		Relational	Relationship with the team members	Trust: ‘…team harmony is formed when there is mutual trust…’ Respect and professional recognition: ‘…be seen as professionals and then be heard…’
			Relationship with users and/or caregivers	‘…know how to empathise with patients…’
			Professional autonomy	‘…you are alone’
			Leadership	Global management of the scenario ‘…no need to talk, some things are already obvious’.
		Opportunities for professional development		‘…I've trained a lot, I've done many courses, I can't wait to test myself…’

The quantitative analysis was conducted using Jamovi 2.3.28 software (Jamovi Project 2023). The adapted tool, called PHE‐ProQoL, demonstrated good content validity, with a Content Validity Index of 0.9 (see Table 2) and strong face validity.

**TABLE 2:** Content validity.

	Expert 1	Expert 2	Expert 3	Expert 4	Expert 5	Expert 6	Expert 7	I‐CVI
Item 1	1	1	1	1	1	1	1	1
Item 2	1	1	1	1	1	1	1	1
Item 3	1	1	1	1	1	1	1	1
Item 4	1	1	1	1	1	1	1	1
Item 5	1	1	1	0	1	1	1	0.86
Item 6	1	1	1	1	1	1	1	1
Item 7	1	1	0	1	1	1	1	0.86
Item 8	1	1	0	0	1	1	1	0.71
Item 9	0	1	0	1	1	1	1	0.71
Item 10	1	1	1	1	1	1	1	1
Item 11	1	1	1	1	1	1	1	1
Item 12	1	1	1	1	1	1	1	1
Item 13	0	1	1	1	1	1	0	0.71
Item 14	1	1	1	1	1	1	1	1
Item 15	0	1	1	0	1	1	1	0.71
Item 16	1	1	1	1	1	1	1	1
Item 17	1	1	1	1	1	1	1	1
Item 18	1	1	1	1	1	1	1	1
Item 19	0	1	1	0	1	1	1	0.71
Item 20	1	1	1	1	0	1	1	0.86
Item 21	1	1	0	1	1	1	1	0.86
S‐CVI								0.904

An Exploratory Factor Analysis (EFA) was performed to explore the underlying structure of the tool. The results confirmed the presence of three components, consistent with the original instrument. Cronbach's *α* coefficient was calculated for each subscale to assess reliability, demonstrating excellent reliability, with values ranging from 0.88 to 0.94.

The EFA, conducted on a set of 30 items, examined the internal factor structure of the PHE‐ProQoL scale. The Kaiser‐Meyer‐Olkin (KMO) test yielded a value of 0.80, indicating that the sample size was adequate for the analysis (Kline 2014). Multiple criteria, including the Kaiser criterion (eigenvalues > 1), scree plot, parallel analysis and minimum average partial (MAP) test, all supported the extraction of three factors. These three factors aligned with the conceptual basis of the PHE‐ProQoL. An oblique (promax) rotation was applied to the three‐factor solution, and items were removed based on established factor loading criteria. This process was repeated twice to ensure that no further items needed removal. Ultimately, 9 items were eliminated, resulting in a final structure of 40 items across three factors.

The overall reliability of the tool was high, with a Cronbach's *α* coefficient of 0.97, indicating excellent internal consistency. All subscales showed strong reliability values, with ‘Compassion Satisfaction’ and ‘Burnout’ reporting a Cronbach's Alpha of 0.941, while ‘Post‐Traumatic Stress’ showed a slightly lower value of 0.883.

### 4.2 Study Results

The study sample consisted of 89 pre‐hospital emergency nurses, with a majority being male (*n* = 46, 51.7%). The average age of the participants was 48.1 years (SD = 8.29). These nurses had an average of 23.5 years (SD = 9.72) of overall professional experience, with 13 years (SD = 3.24) specifically in the pre‐hospital emergency service.

The assessment of EI using the Brief Emotional Intelligence Scale indicated a medium‐high level of EI among the participating nurses. The findings showed that these nurses were generally more skilled at assessing and regulating the emotions of others than managing their own emotions (Table 3).

**TABLE 3:** Emotional intelligence in participants.

Brief emotional intelligence scale‐10 (BEIS‐10)	Mean [min 10–max 50]	SD	Confidence interval
Total score	28.0	±9.58	26–30.1
Factor 2 ‘Evaluation of others' emotions’	5.44	±2.04	5.01–5.87
Factor 3 ‘Regulating one's own emotions’	4.88	±2.15	4.42–5.33
Factor 4 ‘Regulation of others' emotions’	6.88	±2.17	6.42–7.33
Factor 5 ‘Utilisation of emotions’	5.79	±2.50	5.26–6.31

Regarding the PHE‐ProQoL scale, the results showed a medium level of compassion satisfaction among participants, with scores ranging from a minimum of 10 to a maximum of 50. However, there were concerning levels of burnout (range 6–30) and post‐traumatic stress (range 5–25) among the nurses (see Table 4).

**TABLE 4:** Professional quality of life in participants.

Pre‐hospital emergency professional quality of life (PHE‐ProQoL)	Mean [min 10–max 50]	SD	Confidence interval
Factor 1 ‘Compassion satisfaction’	27.3	±9.81	25.3–28.4
Factor 2 ‘Burnout’	22.5	±6.09	21.3–23.8
Factor 3 ‘Secondary traumatic stress’	21.2	±4.3	20.3–22.1

Further analysis examined the correlation between EI and the three factors related to professional well‐being (compassion satisfaction, burnout and post‐traumatic stress). Statistically significant negative correlations were found between EI and both burnout and post‐traumatic stress. Specifically, as EI increased, burnout decreased (Pearson's *r* = −0.859, *p* < 0.001), and post‐traumatic stress also decreased (Pearson's *r* = −0.792, *p* < 0.001). In contrast, a strong positive correlation was observed between EI and compassion satisfaction, indicating that compassion satisfaction increased as EI rose (Pearson's *r* = +0.917, *p* < 0.001).

## 5 Discussion

This study highlights the crucial role of EI in promoting occupational well‐being among pre‐hospital emergency nurses, consistent with existing literature on its protective effects. The findings demonstrate a strong, statistically significant negative correlation between EIand both burnout and post‐traumatic stress, which aligns with previous studies that identify EI as a buffer against these stressors (Năstasă and Fărcaş 2015; Nightingale et al. 2018). Additionally, a strong positive correlation was observed between EI and compassion satisfaction, further underscoring the beneficial impact of EI on professional well‐being (Maillet and Read 2021).

The results indicate that pre‐hospital emergency nurses generally possess moderate levels of compassion satisfaction and exhibit a notably high level of EI, particularly in perceiving and managing others' emotions. However, these positive attributes coexist with a significant prevalence of burnout and post‐traumatic stress, reflecting the demanding nature of their work environment (Smith 2017). This duality points to the complex emotional dynamics within pre‐hospital emergency settings, where high EI helps mitigate some of the adverse effects associated with stressors inherent in emergency care.

These findings emphasise the need for targeted interventions to enhance emotional resilience among emergency care professionals. Integrating EI training into healthcare education and professional development programmes could play a pivotal role in fostering a supportive work environment, ultimately benefiting both caregiver well‐being and patient care quality (Thompson and Gullone 2020). Moreover, enhancing EI can equip nurses with better tools for coping with stress, improving communication and fostering teamwork, all critical for optimal performance in high‐pressure settings (Johnson and Smith 2019).

The study also highlights the utility of the adapted ProQoL scale for the pre‐hospital emergency context. Validating this tool for use in emergency settings provides healthcare managers with a reliable method to assess and address the well‐being of their staff. This adaptation is crucial given the unique challenges faced by pre‐hospital emergency nurses, who often operate in unpredictable environments with limited resources. A validated tool enables more precise monitoring of their professional quality of life, guiding interventions that promote health and resilience in these challenging roles.

In light of these insights, future research should focus on developing and implementing EI‐based interventions tailored to the specific challenges of pre‐hospital emergency settings. Further studies could explore the longitudinal impact of EI training on professional well‐being and patient outcomes, providing a deeper understanding of how these strategies can be optimised to enhance both nurse retention and care quality.

Overall, this study contributes to the growing body of evidence supporting the integration of EI into the training and ongoing professional development of pre‐hospital emergency nurses. It calls for a more comprehensive approach to managing occupational health, where fostering emotional skills becomes as fundamental as developing clinical expertise.

By addressing the unique demands of the pre‐hospital emergency context, the findings promote a holistic strategy that enhances the well‐being of emergency care professionals and supports the overall effectiveness and sustainability of emergency services. This underscores the value of continued research and investment in EI as a key component of professional health and resilience in healthcare settings.

## 6 Conclusions

Given the complex demands in healthcare, prioritising the well‐being of healthcare professionals is essential not only to mitigate job dissatisfaction and attrition rates but also to maintain the quality of patient care. The introduction of the Pre‐Hospital Professional Quality of Life tool represents a significant advancement, providing a consistent means to evaluate and enhance the professional quality of life of pre‐hospital emergency nurses. Central to this effort is the enhancement of EI, which is increasingly recognised as a cornerstone in bolstering the overall professional health of nursing staff. The competencies involved in EI, such as empathy, self‐awareness and emotional regulation, are indispensable, paralleling the importance of academic knowledge and technical proficiency in nursing practice.

Empirical evidence supports the tangible benefits of EI training in improving professional health, reinforcing the need to embed such training within foundational nursing education and ongoing professional development programmes (McQueen 2004). These programmes should cultivate not only emotional acuity but also leadership skills, preparing nurses to navigate and excel in the multifaceted healthcare landscape. Furthermore, the nursing recruitment process should incorporate the evaluation of emotional competence, recognising its critical role in ensuring quality care and fostering a supportive team environment (Akerjordet and Severinsson 2008).

It is also crucial to acknowledge the lack of research focused specifically on the pre‐hospital emergency context. This sector, distinct in its dynamics and challenges from other critical care environments, warrants a tailored investigative approach to uncover and address its unique needs. Highlighting these aspects will not only enrich the academic discourse but also pave the way for targeted interventions and policies, ultimately enhancing the professional quality of life and operational efficiency in pre‐hospital emergency services.

## 7 Recommendations

While this study advocates for the integration of EI training in nursing education and professional development, it also highlights the need for further research. Future studies should focus on validating these findings with larger and more diverse samples and exploring the broader impact of EI on various aspects of nursing performance and patient care outcomes. Additionally, the effectiveness of implementing EI training programmes should be tested for their potential to reduce burnout, enhance job satisfaction and improve overall workplace culture in pre‐hospital emergency settings.

Building on the current evidence, future efforts can ensure that EI becomes a cornerstone of nursing practice, promoting both individual well‐being and systemic resilience in healthcare environments.

## 8 Limitations

This study has several limitations that should be addressed in future research. The use of a convenience sample limits the generalisability of the findings, as the sample may not represent all pre‐hospital emergency nurses. Additionally, the EFA performed in this study suggests the need for further research, including confirmatory factor analysis, to fully validate the scale. Future studies should consider larger and more diverse samples, as well as longitudinal designs, to better understand the dynamic relationship between EI and professional well‐being. Furthermore, the use of self‐reported measures might introduce bias, and future studies should aim to include more objective measures of EI and related outcomes.

In conclusion, while this study provides valuable insights into the impact of EI on professional well‐being among pre‐hospital emergency nurses, more comprehensive research is needed to expand upon these findings and develop targeted interventions that support the well‐being of healthcare professionals globally.

### Conflicts of Interest

The authors declared no potential conflict of interest with respect to the research, authorship and/or publication of this article.

## Supporting information


Data S1.

## Data Availability

The data that support the findings of this study are available from the corresponding author upon reasonable request.
